# Role of valves in non-segmented flow systems

**DOI:** 10.1155/S1463924687000063

**Published:** 1987

**Authors:** A. Ríos, M. D. Luque de Castro, M. Valcárcel

**Affiliations:** Department of Analytical Chemistry Faculty of Sciences University of Córdoba Córdoba Spain


Journal of Automatic Chemistry/*Journal of Clinical Laboratory Automation*, Vol. 9, No. 1 (January–March 1987), pp. 30–36

### Role of valves in non-segmented flow systems

**A. Ríos, M. D. Luque de Castro and M. Valcárcel**

*Department of Analytical Chemistry, Faculty of Sciences, University of Córdoba, Córdoba, Spain*

Valves are essential components in non-segmented flow systems, particularly in Flow Injection Analysis (FIA). Although their usual role is associated with sample 'injection' into a carrier stream, this is not by any means their sole use. In hydrodynamic systems, valves must also be regarded as components allowing the manipulation of the flow and affording a high degree of automation of the operation or function they are to carry out. Valves permit simple alterations in the flow-rate to be performed, as well as fully controlling sample treatment (chemical reaction etc.). Sometimes, by coupling the two functions (injection and manipulation of the different flow-rates) extremely difficult problems can be solved.

The different types of valves available can be classified according to their performance into the three groups listed in table 1; this includes their potential functions. While rotary valves switch between various streams by a simple turn, commutation valves work by sliding one of the parts with respect to the others. Solenoid valves are more sophisticated and their operation (circulation of the sample through the system for a preselected time) is electronically controlled. As it is sometimes of interest to automate these elements with a minicomputer, the classification shown in table 1 is important because the different categories of interfaces needed in each case is related to their type of movement (or time control).

### Injection valves

Injection valves introduce the sample into the flow system. The term 'injection' is an old-fashioned one, referring to the early systems described by Ruzicka and Hansen [1 and 2], which are based on the use of a needle-less syringe, whose plunger presses and displaces a septum, thus creating a transient chamber which holds the sample. As the septum returns to its original position, the sample is introduced into the carrier solution. These systems were described by Betteridge in a review published in 1978 [3]. A syringe with a hypodermic needle injecting the sample into the carrier through a septum has also been used. Injection proper results in a transient change in the flow which yields a sharp and irreproducible peak. These systems have been superseded by valves and commutator, which allow accurate and reproducible volumes of the sample solution to be inserted (rather than injected) with no disturbance to the flow, the injected volume varying over a wide range. Their easy and fast handling allows their operation to be automated.

*Rotary valves*

Rotary valves are commonly used to introduce the sample into the system and are usually four-way valves with two inlet ports (carrier and sample solutions) and two outlet ports, in addition to a variable-volume loop which is filled with the sample. There is a total of six channels: three inlets and three outlets. Sample insertion is achieved by turning the cylinder on the support, which allows the inlet and outlet ports to be connected by means of three hacks bored on the upper tape of the cylinder [4] (figure 1 [*a*]). The commercial injection valves used in analysers like FIAstar, the loop of which is filled by means of a syringe [5 and 6], are very similar. In general, the rotary valves used in FIA are similar to those employed in HPLC, although the former can be constructed from cheaper materials as they do not have to withstand high pressures.

Rotary injection valves are sometimes moved pneumatically to co-ordinate their operation with that of a sample tray by means of a microprocessor [7 and 8]. A high degree of automation is achieved in this way and the systems have features typically found in auto-analysers. Jørgensen *et al.* use pneumatically operated valves to control analytical sensitivity, both in the introduction of the sample and for pre-concentration by ion exchange [9].

*Table 1. Classification of the different types of valves used in non-segmented flow systems.*

| Type of valve | Functions |
|---|---|
| (*a*) Rotary<br>With four-channels | Selection or transport of the different channels.<br>Mixing of two channels and selecting the direction of the resulting channel.<br>Split of the incoming channel. |
| With six-channels | Sample injection.<br>Channel selection (with waste).<br>Selection-diversion.<br>Rough modification of the flow-rate. |
| (*b*) Commutation<br>With three channels | Selection-diversion (without waste).<br>Stopped-flow. |
| With more than three channels | Selecting one channel from several.<br>Simultaneous confluence of several channels with one or several outlets. |
| (*c*) Solenoid | Sample injection. |

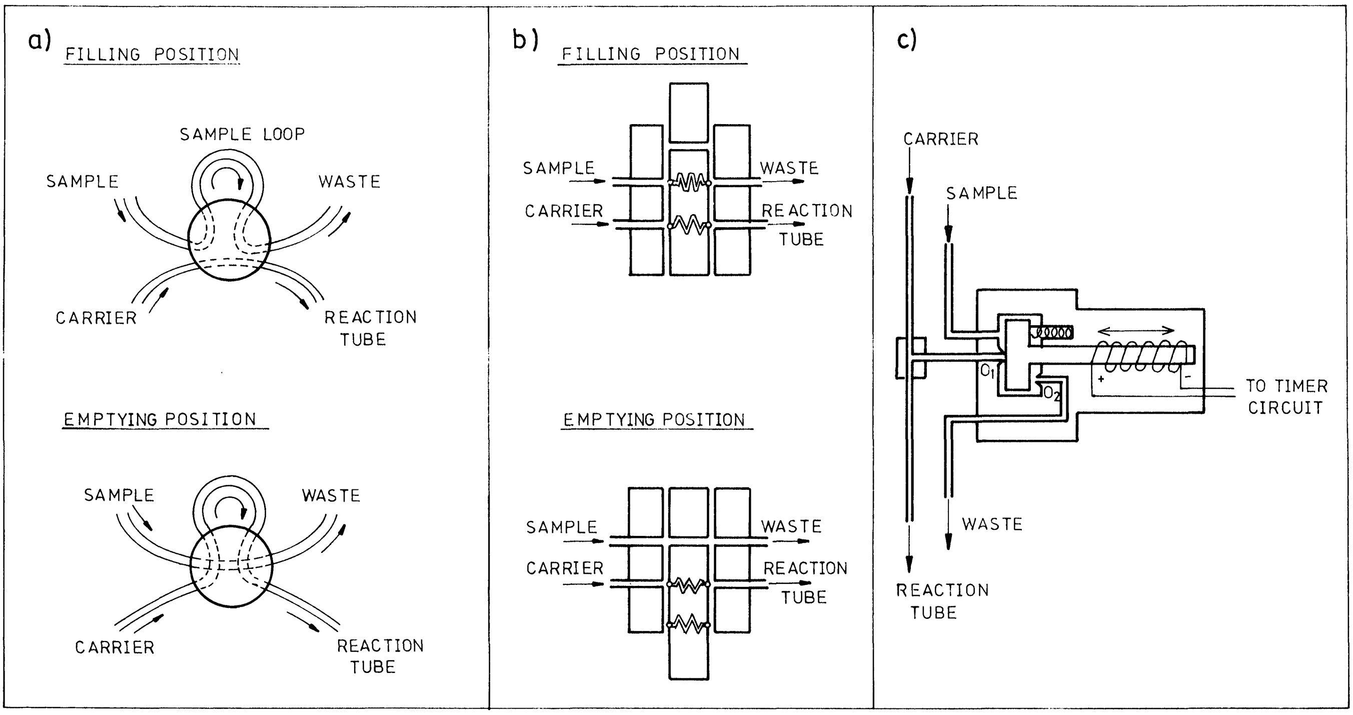

*Figure 1. Different systems for sample insertion in non-segmented flow configurations. (a) rotary valve; (b) proportional injector; (c) solenoid valve controlled by timer circuit (the figure corresponds to the filling position: $O_1$ closed and $O_2$ open. Sample insertion: $O_1$ open and $O_2$ closed).*

Dual rotary valves have only been used to insert samples or a sample and a reagent (merging zones) simultaneously into the flow system. This injection system is built by coupling two ordinary single rotary valves [4] and in the past a shortcoming was a fixed injection volume (this drawback has now been solved [10 and 11]).

*Proportional injectors*

Although they are not strictly injection valves, the systems developed by a Brazilian FIA team are of interest here [12]. These are based on commutation principles and introduce samples (single or multiple) into the FIA system, as well as performing other hydrodynamic functions. Proportional injectors insert one or several volumes of sample and/or reagents simultaneously into the flow system by sliding the central block with respect to the two side blocks, which remain fixed (figure 1 [*b*]). The central block can be in either of two positions (filling and emptying). The proportional injector is highly versatile and allows most FIA modes to be used [13].

*Solenoid valves*

Solenoid valves are more complicated, electronically controlled injection systems. Although their chief purpose is the insertion of fixed or variable sample volumes into the flow system they also allow various FIA modes to be used. These valves were once included in such commercial analysers as FIAtron's SHS 200.

Rothwell and Woolf have proposed a solenoid valve with a timer circuit as an ideal element for flow systems [14]. They consider that this valve (figure 1 [*c*]) has several advantages over the ordinary rotary valve: it is cheaper and the injected sample volume can be continuously changed as it is manipulated by temporal rather than spatial control as in rotary valves.

*Other alternatives*

There are ways of introducing the sample into the flow system which do not use injection valves. Thus, the 'hydrodynamic injection' described by Ruzicka and Hansen [15], requires a timer to control the stop-and-go of two pumps (one for the carrier solution channel and another for the sample channel, which are perpendicular to each other and share a tubing zone whose volume is that of the inserted sample). The intermittent operation of one or other pump corresponds to the filling and emptying positions. This injection system has several disadvantages which are not compensated for by the cost-savings involved in avoiding the use of an injection valve.

A more interesting alternative is that suggested by Riley *et al.* [16], which uses an intermittent pump instead of an injection valve. It consists of a mobile probe connected to the tube of the peristaltic pump and aimed to aspirate small sample volumes which are periodically introduced into the flow system. The motion of the probe and the pump is strictly controlled by a microcomputer through a step motor.

In both modes, the elements required to replace the injection valve result in a more complex system, making them unsuitable alternatives to units above.

### Diverting-selecting valves

This type of valve allows an outgoing channel to be separate from several incoming ones. Alternatively, an incoming channel can be driven to a point between several others. The overall FIA system requires the presence of an injection valve or one of the units described above – except in the case of completely continuous flow systems [17 and 18]. The chief use of diverting-selecting valves is to modify the hydrodynamic conditions of the system at a pre-selected time. Some types of commutators perform both functions (injection/selection-diversion) in a single unit [13].

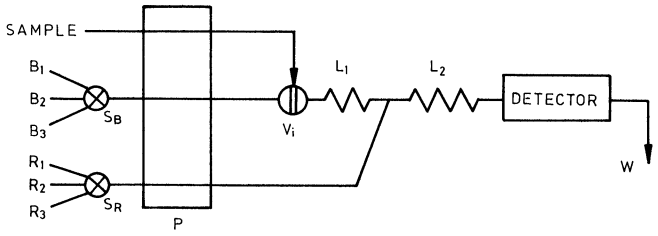

*Figure 2. Use of commutation valves with more than three ways, $S_B$ and $S_R$, for selection of buffer (B) and reagent (R) in simultaneous determinations ($V_i$ = injection valve; L = reactor; P = pump; W = waste).*

*With commutation valves*

These are the most simple systems used to select or divert one or several channels. Their salient feature is the existence of one or several switches governing two alternative opening and closing channels.

Three-way or simple commutation valves have a single switch and three channels: the central one is always open and communicates with one of the side channels in line with the position of the switch. This unit can perform two different functions: (1) selection of an outgoing channel from two incoming ones; and (2) diversion of an outgoing stream to two different outgoing channels. As this system has no waste channel, for function (1) the valve must be located behind the propulsion system, and after the pump in function (2). Its greatest advantage lies in the simplicity with which it can be incorporated into the flow configuration, allowing the nature of the selected channel to be instantly and drastically changed (but not the flow-rate), or driving this to two different positions. In this last case, the flow of one of the outgoing channels is stopped, so these valves can also be used to develop stopped-flow techniques. When the reacting plug is at the detector the channel of the carrier solution is driven to waste and the flow in the other outgoing channel of the

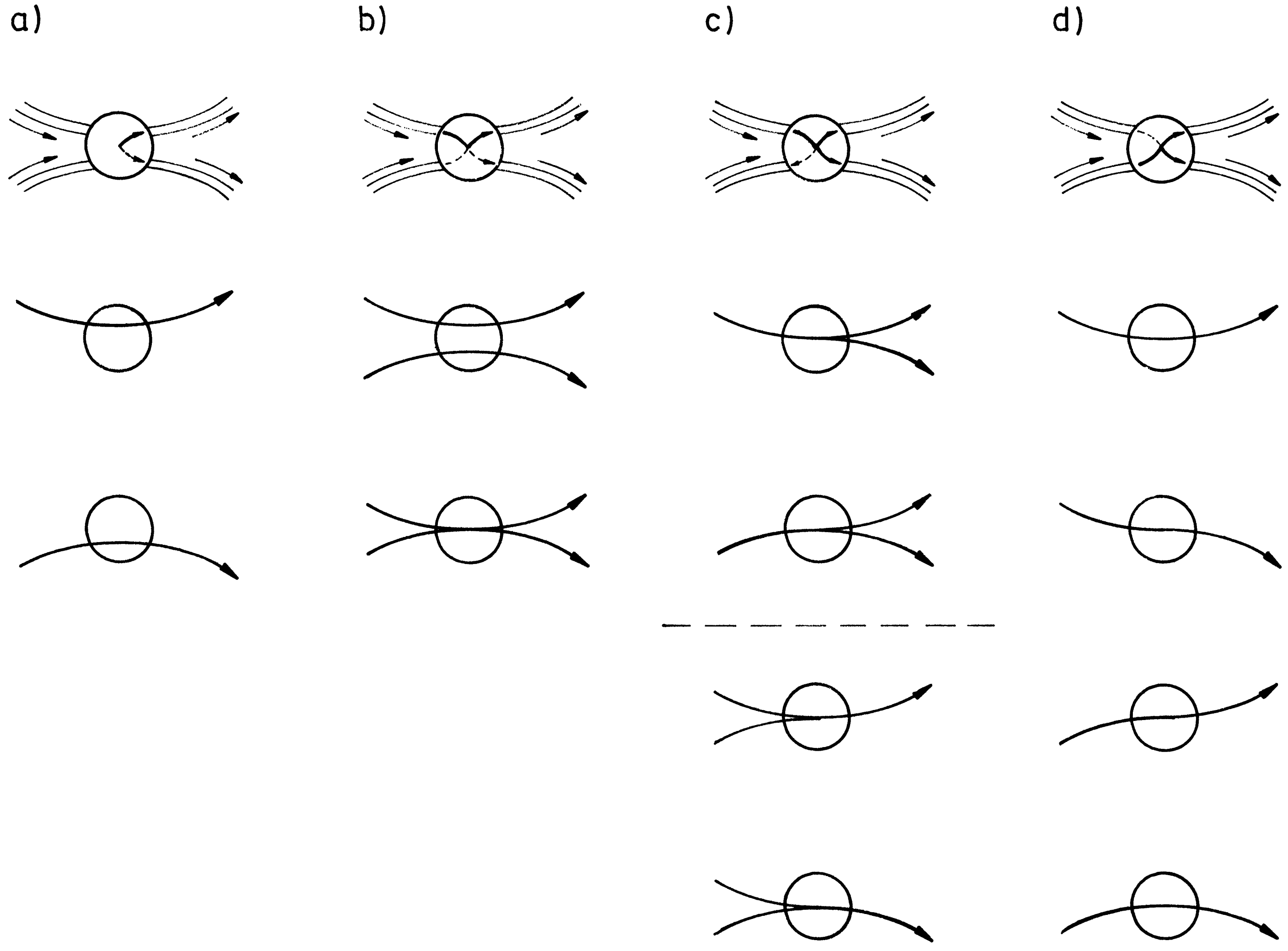

*Figure 3. Main function of four-way rotary valves in flow-systems: (a) and (b) with two rotary positions; (c) with two rotary positions and two possible types of connection (splitting or confluence of the flow); (d) with four positions.*

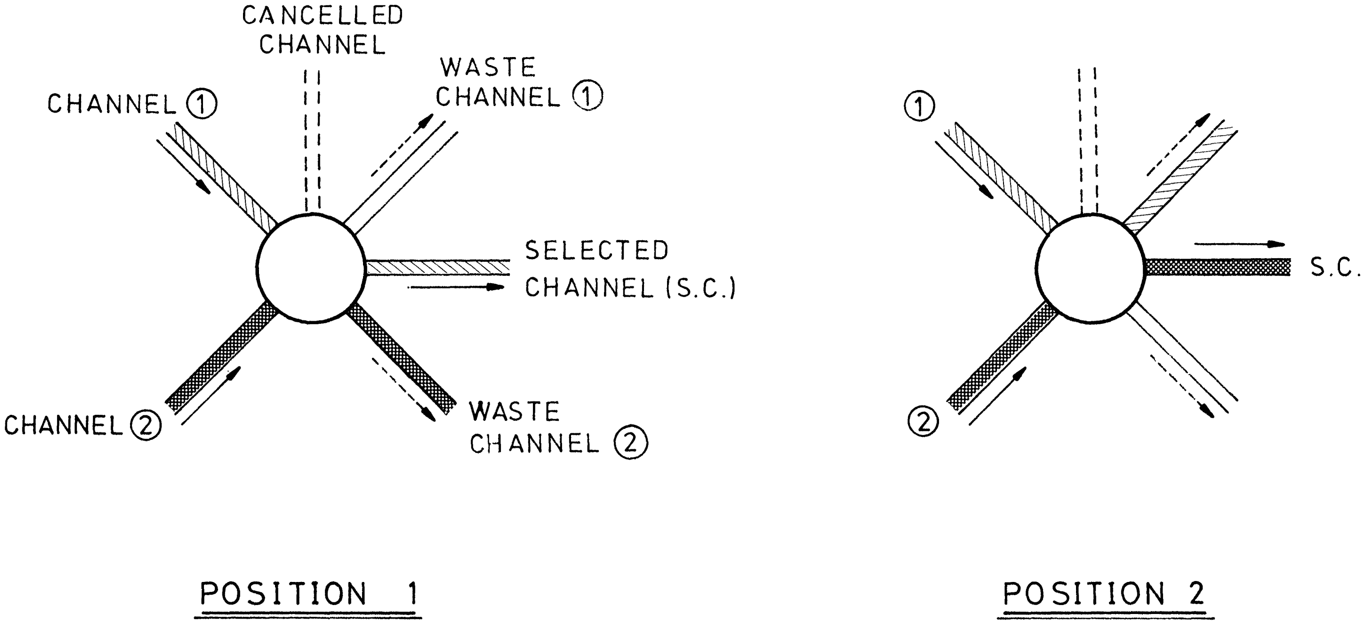

*Figure 4. Use of an ordinary six-way injection valve as a selecting valve.*

valve remains stopped during the time over which the switch is in this position.

Most three-way commutation valves (multiple commutation valves) have more than one switch (as many as channels they have). Each switch acts on a single channel, opening or closing it by turning the commutation element (small Teflon key). This type is the most suitable for selecting a given channel, and it is usually placed before the propulsion system. It is especially useful for determining two or more parameters and where individual determinations have to be carried out at a different pH, with different reagents and/or in the presence of different masking agents [19]. Thus, with a simple configuration (figure 2) and with the aid of two of these valves, three parameters in a sample can be sequentially determined. Valve S in figure 2 selects the suitable streams (buffer + masking agents) and reagent before sample injection.

Rock *et al.* use one of these valves with four outlets located before the pump to divert the carrier solution, stopping the flow sequentially in the four channels and prior to their confluence before reaching the detector [20]. The sample is introduced into the carrier solution containing the reagent by intermittent pumping [16]. The main advantage of this configuration is a four-fold increase in the sampling frequency.

Commutation valves with more than three channels can also be used for the simultaneous confluence of two or more channels with one or several outlets.

*With rotary valves*

These systems allow the selection or diversion of the channels by turning a cylinder placed on a support, thus offering a wider range of functions than commutation valves. The mobile cylinder can be switched between two or more positions.

Four-way rotary valves, whose uses are illustrated in figure 3, are designed exclusively for hydrodynamic manipulation of the flow system. Their use in FIA has been very limited, but they have enormous potential: channel selection, diversion, splitting and confluence, with the option of selecting one of the two incoming and two outgoing channels. Most of them have two positions (*a*, *b* and *c* in figure 3) and some, such as *c*, can adopt two different situations in the flow systems. One position permits the splitting of the flow into any of the selected channels and the other allows the confluence of two channels and diversion to one of the two outlets. The valve can also be switched to four different selecting-diverting positions (figure 3 [*d*]).

The six-way rotary valves usually employed in injection systems can also be adapted to selecting-diverting valves by an alternative connection of the different channels, as shown in figure 4 [19]. One of the channels is not in use and of the other five, two are incoming channels and three outgoing channels (one of which is always inactive). Of the two incoming channels one is selected and the other is waste through one of the outlets. Valves thus connected offer such novel uses (figure 5) as co-ordination of two FIA subsystems selecting the flow coming from one of them by its location prior the detector (figure 5 [*a*]). This configuration is especially useful for simultaneously monitoring different parameters in the same sample this could otherwise require very different manifolds [19].

A simpler use of these valves is the rough alteration (an increase) of the flow-rate, resulting in a washout effect which allows sampling frequency to be increased without the use of alternating streams [21] or flow gradients [22] (figure 5 [*b*]). The fact that each stream attaining the valve is wasted through a different channel endows these valves with a selecting-diverting capability so that any of the channels driven to waste can attain any point in the FIA configuration (figure 5 [*c*]) (as used in speciation studies [23]).

This valve is the key to designing open-closed systems (figure 5 [*d*]) in which multidetection is carried out by the iterative passage of the reacting plug through a single detector [24 and 25]. In this case, one of the channels attaining the valve is the carrier solution and the other is that coming from the detector. In the open position, valve S selects the carrier solution and drives it to the detector, after which it is wasted. In the closed-system position a closed circuit is established, as a result of valve S selecting the channel coming from the detector. In this last position the evolution (physical and chemical) of an injected sample into the flow can be monitored.

Olsen *et al.* [26] also use a configuration with three six-way rotary valves as directional valves in a two-line FIA-AAS system to determine levels of heavy metals in sea water. The authors couple a Chelex-100 ion-exchange column for pre-concentration. There are two cycles fixed by the position of these valves: in the pre-concentration cycle, the injected sample volume (2 ml) passes through the column, the metal ions being retained; in the elution cycle (use of 2M $HNO_3$ as eluent) these ions are driven to the detector. The most important feature of this configuration is that the sample matrix never attains the detector, but only the $HNO_3$ solution (before passing

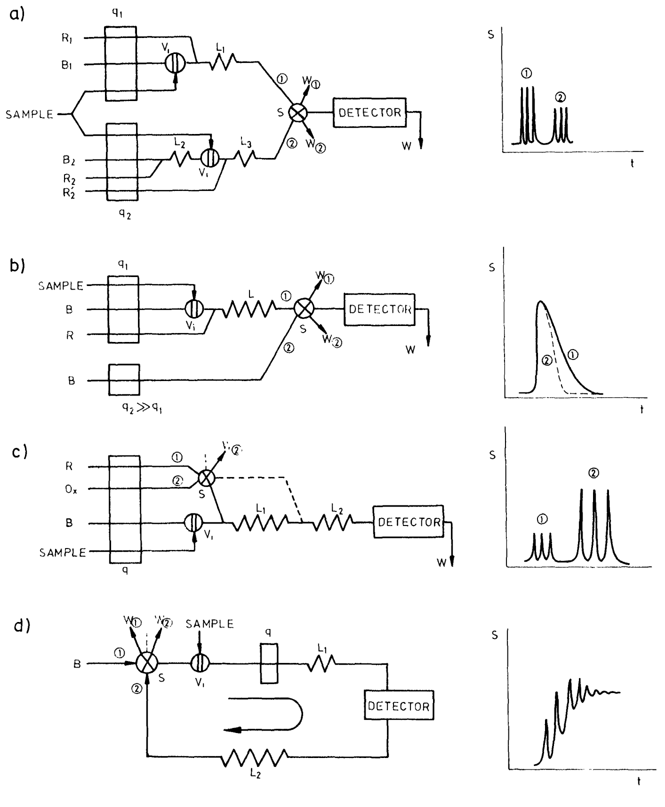

*Figure 5. Various uses of six-way rotary injection valves acting as selecting devices (S) in flow systems (for details, see text). $V_i$ = injection valve; L = reactor; q = flow-rate; R = reagent; B = buffer or carrier solution; Ox = oxidant; W = waste.*

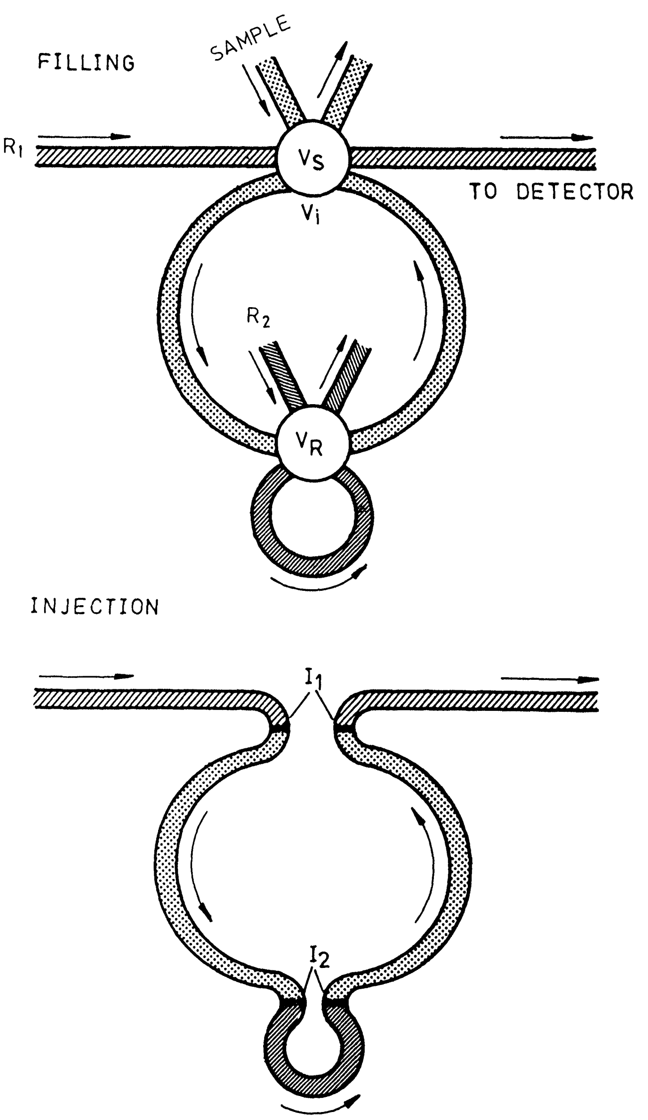

*Figure 6. Internal coupling of two injection valves to establish different reaction zones (interfaces $I_1$ and $I_2$) in the same plug. (a) filling of the sample, $V_S$, and regent, $V_R$, valve loop; (b) simultaneous injection of both valves ($R_1$ and $R_2$ can be two different reagents or a given one under different chemical conditions).*

through the column or not) attains it, thus avoiding the background effect. Nevertheless, this configuration can be simplified by replacing the three-valve system by a single six-way injection valve with the ion-exchange column inserted in its loop [27]. The position of this valve should establish pre-concentration-elution cycles and the same carrier stream ($HNO_3$) should always reach the detector.

### Valve coupling

Some of the configurations described above use more than one valve, usually with different functions. Nevertheless, such valves are not coupled because they do not operate simultaneously or follow a defined sequence to fulfil a single function, but one establishes the chemical or hydrodynamic conditions required for the operation of the other. Valves to be coupled will be assumed to be meant for the same purpose.

*Serial coupling*

There are several ways of serial coupling, for example serial sequential injection of several selective reagents for two or three analytes into the same sample, which acts as a carrier solution (reversed FIA mode) have been used for photometric determination of pollutants in waste water [28]. A set of three peaks, overlapping or not, are obtained per analysis. Peak height is related to the concentration of each analyte (a calibration curve for each is previously run).

Serial simultaneous injection of the sample into a reagent carrier has also been used by a Brazilian team [29] as a more accurate alternative to gradient dilution techniques [30]. A proportional injector was used to insert three sample bolusses into the carrier thus yielding three overlapping peaks on passage through the detector, whose decreasing height is a result of increasing dispersion. This alternative allows the analyte to be determined over a wide concentration range (dilution method) because there are three calibration curves with different sensitivity and concentration range obtained from each simultaneous injection. Other interesting applications of the serial simultaneous injection are the kinetic determination of individual species [31] or mixtures [32 and 33] based on the different residence time of the two peaks obtained per injection.

*Parallel coupling*

This configuration has been used by Slanina *et al.* for the determination of certain species in rain-water [34]. Each valve injects the sample into a distilled water channel, later merging with a suitable reagent prior to the multichannel spectrophotometer which receives the parallel channels and is controlled by a computer. The system selecting the standard is also automatized through the computer, which also acts on the coupled valve system (a six-way pneumatically driven valve and an ordinary three-way one).

Undoubtedly, the most common FIA mode making use of parallel injection valves is the merging zone mode [35]. In the symmetric merging zones, sample and reagent are injected into two parallel channels and the bolusses merge symmetricaly prior to the detector. This mode features small sample and reagent consumption, so that it is the most suitable for clinical analysis. Its association with other techniques such as stopped-flow [36] further increases its potential. In the asymmetric mode, one of the injected bolusses merges in the tail of the other bolus, giving rise to a new bolus with two different reaction zones. This mode is the most suitable for such simultaneous determinations as the speciation of chromium is waste water with diphenylcarbazide and cerium(IV) [37].

*Internal coupling*

Internal coupling involving the direct connection of two or more valves to perform a given function. In this area, the coupling of the microcomputer-controlled four-way valves has been proposed to synchronize their operation with that of the sample tray [38] thereby achieving the automatic determination of $CO_2$ in plasma. Other interesting uses of two coupled six-way rotary valves have been suggested by Harnly and Beecher to build an injector which minimizes the 'memory' of the nebulizer in FIA-AAS [39]. The function of the first valve is to insert the sample, while the second valve (directly connected in series with the first) gives rise to washout cycles between successive samples.

A useful alternative to carrying out the pH gradient techniques proposed by Betteridge and Fields [40 and 41] involves the internal coupling of two injection rotary valves (figure 6), one of which is located in the loop of the main one, which is placed in the reagent stream at a given pH. The sample fills the loop of the main valve, while the secondary valve is filled with reagent solution at a pH different from that of the carrier stream. The simultaneous injection of both valves yields four sample-reagent interfaces with two reaction zones (central and terminal) of different pH. In this way, analytes reacting with the same reagent at different pHs (lead-vanadium with pyridilazo resorcinol, PAR [42] and hydrazine-ammonia with o-phtalaldehyde and mercaptoethanol [43]) can be easily determined.

### Conclusions

The most important uses of different valves in non-segmented flow systems have been considered. Using valves as simple sample injection units [44] is just one of their possible functions, because they also allow the manipulation of the hydrodynamic conditions of the flow systems for selecting or deviating channels, stopping the flow, set-up of open-closed systems, etc.

These valves can either be operated manually or they can be automated by electronic timer or by a microcomputer (computer interface[s]), synchronized to a sampler tray.

### References

1. Ruzicka, J. and Hansen, E. H., *Analytica Chimica Acta*, **78** (1975), 145.
2. Stewart, J. W. B., Ruzicka, J., Bergamin, H. F° and Zagatto, E. A. G., ibid., **81** (1976), 371.
3. Betteridge, D., *Analytical Chemistry*, **50** (1978), 832A.
4. Valcárcel, M. and Luque de Castro, M. D., *Flow Injection Analysis: Principles and Applications* (Ellis Horwood Ltd, Chichester, 1986).
5. Hansen, E. H. and Ruzicka, J., *Journal of Chemical Education*, **56** (1979), 677.
6. Ruzicka, J., Hansen, E. H. and Ramsing, A. V., *Analytica Chimica Acta*, **134** (1982), 55.
7. Brown, J. F., Stewart, K. K. and Higgs, D., *Journal of Automatic Chemistry*, **3** (1981), 182.
8. Koupparis, M. and Anagnostopoulou, P., ibid, **6** (1984), 186.
9. Jorgensen, S. S., Petersen, K. M. and Hansen, L. A., *Analytical Chimica Acta*, **169** (1985), 51.
10. Mindegaard, J., ibid, **104** (1979), 185.
11. Lázaro, F., Luque de Castro, M. D. and Valcárcel, M., *Analytical Chimica Acta* (in press).
12. Bergamin, H. F°, Medeiros, J. X., Reis, B. F. and Zagatto, E. A. G., ibid, **101** (1978), 9.

13. Krug, F. J., Bergamin, H. F° and Zagatto, E. A. G., ibid, **179** (1986), 103.

14. Rothwell, S. D. and Woolf, A. A., *Talanta*, **32** (1985), 431.

15. Ruzicka, J. and Hansen, E. H., *Analytical Chimica Acta*, **145** (1983), 1.

16. Riley, C., Aslett, L. H., Rocks, B. F., Sherwood, R. A., McK.Watson, J. D. and Morgan, J., *Clinical Chemistry*, **29** (1983), 332.

18. Goto, M., *TrAC Trends Anal. Chem.*, **92** (1983).

19. Ríos, A., Luque de Castro, M. D. and Valcárcel, M., *Talanta*, **31** (1984), 673.

20. Rocks, B. D., Sherwood, R. A., Hosseinmardi, M. M. and Riley, C., *Analytica Chimica Acta*, **179** (1986), 225.

21. Ruzicka, J. and Hansen, E. H., *Flow Injection Analysis* (Wiley & Sons, New York, 1981), 72.

22. Ríos, A., Luque de Castro, M. D. and Valcárcel, M., *Talanta*, **32** (1985), 845.

23. Ruz, J., Ríos, A., Luque de Casto, M. D. and Valcárcel, M., *Analytica Chimica Acta*, **186** (1986), 139.

24. Ríos, A., Luque de Castro, M. D. and Valcárcel, M., *Analytical Chemistry*, **57** (1985), 1803.

25. Idem, *Analytical Chimica Acta*, **179** (1986), 463.

26. Olsen, S., Pessenda, L. C. R., Ruzicka, J. and Hansen, E. H., *Analyst*, **108** (1983), 905.

27. Cañete, F., Ríos, A., Luque de Castro, M. D. and Valcárcel, M., *Analyst* (in press).

28. Ríos, A., Luque de Castro, M. D. and Valcárcel, M., *Analyst*, **109** (1984), 1487.

29. Zagatto, E. A. G., Giné, M. F., Fernandes, E. A. N., Reis, B. F. and Krug, F. J., *Analytica Chimica Acta*, **173** (1985), 289.

30. Olsen, S., Ruzicka, J. and Hansen, E. H., ibid, **136** (1982), 101.

31. Fernández, A., Luque de Castro, M. D. and Valcárcel, M., *Quím. Anal.* (in press).

32. Idem, *Analytica Chimica Acta* (in press).

33. Idem, *Analyst* (in press).

34. Slanina, J., Bakker, F., Bruyn-Hes, A. and Möls, J. J., *Analytica Chimica Acta*, **113** (1980), 331.

35. Bergamin, H. F°, Zagatto, E. A. G., Krug, F. J. and Reis, B. F., ibid, **101** (1978), 191.

36. Ruzicka, J. and Hansen, E. H., ibid, **106** (1979), 207.

37. Ruz, J., Ríos, A., Luque de Castro, M. D. and Valcárcel, M., Fresenius, Z. *Anal. Chem.*, **322** (1985), 499.

38. Baadenhuijsen, H. and Seuren-Jacobs, H. E. H., *Clinical Chemistry*, **25** (1979), 443.

39. Harnly, J. M. and Beecher, G. R., *Analytical Chemistry*, **57** (1985), 2015.

40. Betteridge, D. and Fields, B., *Analytical Chemistry*, **50** (1978), 654.

41. Betteridge, D., Dagless, E. L., Fields, B., Sweet, P. and Deans, D. R., *Anal. Proc.*, **26** (1981).

42. Ríos, A., Luque de Castro, M. D. and Valcárcel, M., *Analytical Chemistry*, **58** (1986), 663.

43. Idem, *Analytical Chimica Acta* (in press).

44. Melhik, S. and Fejes, J., *Chem. Listy*, **79** (1985), 910.

### X-RAY SPECTROPHOTOMETRY

A five-day conference on X-ray spectrometry is to be presented by Philips Analytical at the University of Durham in 1987. It will cover advances in instrumentation, interesting applications, methods for qualitative and quantitative analysis, sample preparation, sample robotics and alternative techniques with speakers from instrument manufacturers, industry, educational and research establishments.

Organized jointly by Philips Analytical and Durham University's Department of Geological Sciences, the conference, the 15th in the series, has been booked for 14 to 18 September.

*Further information can be obtained from Maureen Courtney, Pye Unicam Ltd, York Street, Cambridge CB1 2PX, UK. Tel.: 0223 358866.*

### ANALYTICON

The dates for 1987's Analyticon meeting are 12 to 14 October and the venue is London. There will again be 18 sessions running in three concurrent streams. Analyticon 86 topics to be retained (mostly in modified form) will be robotics, laboratory information management systems, sensors, laboratory economics, quality assurance, chemometrics and safety. Education and training in analytical chemistry will be a popular session which will be concerned with the ACOL scheme (Analytical Chemistry by Open Learning).

*The organizers are Scientific Symposia Ltd, 33/35 Bowling Green Lane, London EC1R 0DA.*

